# Molecular Characteristics of IS*1216* Carrying Multidrug Resistance Gene Cluster in Serotype III/Sequence Type 19 Group B Streptococcus

**DOI:** 10.1128/mSphere.00543-21

**Published:** 2021-07-28

**Authors:** Yong Zhi, Hyun Jung Ji, Jong Hyun Jung, Eui Baek Byun, Woo Sik Kim, Shun Mei Lin, Sangyong Lim, A-Yeung Jang, Min Joo Choi, Ki Bum Ahn, Jae Hyang Lim, Joon Young Song, Ho Seong Seo

**Affiliations:** a Research Division for Biotechnology, Korea Atomic Energy Research Institute, Jeongeup, Republic of Korea; b Department of Radiation Science, University of Science and Technology, Daejeon, Republic of Korea; c Department of Oral Microbiology and Immunology, DRI, and BK21 Plus Program, School of Dentistry, Seoul National University, Seoul, Republic of Korea; d Functional Biomaterial Research Center, Korea Research Institute of Bioscience and Biotechnology, Jeongeup, Republic of Korea; e Department of Internal Medicine, Korea University Guro Hospital, Korea University College of Medicine, Seoul, Republic of Korea; f Department of Microbiology, Ewha Womans University College of Medicine, Seoul, Republic of Korea; g Ewha Education & Research Center for Infection, Ewha Womans University Medical Center, Seoul, Republic of Korea; UTMB

**Keywords:** *Streptococcus agalactiae*, IS*1216*, multidrug resistance gene, *srr1/2*, ST19

## Abstract

Streptococcus agalactiae is the leading cause of meningitis in newborns and a significant cause of invasive diseases in pregnant women and adults with underlying diseases. Antibiotic resistance against erythromycin and clindamycin in group B streptococcus (GBS) isolates has been increasing worldwide. GBS expresses the Srr1 and Srr2 proteins, which have important roles in bacterial infection. They have been investigated as novel vaccine candidates against GBS infection, with promising results. But a recent study detected non-*srr1/2*-expressing clinical isolates belonging to serotype III. Thus, we aimed to analyze the genotypes of non-*srr1/2* GBS clinical isolates collected between 2013 and 2016 in South Korea. Forty-one (13.4%) of the 305 serotype III isolates were identified as non-*srr1/2* strains, including sequence type 19 (ST19) (*n* = 16) and ST27 (*n* = 18) strains. The results of the comparative genomic analysis of the ST19/serotype III/non-*srr1/2* strains further revealed four unique gene clusters. Site 4 in the *srr1* gene locus was replaced by an *lsa*(E)-*lnu*(B)-*aadK*-*aac-aph*-*aadE*-carrying multidrug-resistant gene cluster flanked by two IS*1216* transposases with 99% homology to the enterococcal plasmid pKUB3007-1. Despite the Srr1 and Srr2 deficiencies, which resulted in reduced fibrinogen binding, the adherence of non-*srr1/2* strains to endothelial and epithelial cells was comparable to that of Srr1- or Srr2-expressing strains. Moreover, their virulence in mouse models of meningitis was not significantly affected. Furthermore, additional adhesin-encoding genes, including a gene encoding a BspA-like protein, which may contribute to colonization by non-*srr1/2* strains, were identified via whole-genome analysis. Thus, our study provides important findings that can aid in the development of vaccines and antibiotics against GBS.

**IMPORTANCE** Most previously isolated group B streptococcus (GBS) strains express either the Srr1 or Srr2 glycoprotein, which plays an important role in bacterial colonization and invasion. These glycoproteins are potential protein vaccine candidates. In this study, we first report GBS clinical isolates in which the *srr1/2* gene was deleted or replaced with foreign genes. Despite Srr1/2 deficiency, *in vitro* adherence to mammalian cells and *in vivo* virulence in murine models were not affected, suggesting that the isolates might have another adherence mechanism that enhanced their virulence aside from Srr1/2-fibrinogen-mediated adherence. In addition, several non-*srr1/2* isolates replaced the *srr1/2* gene with the *lnu*(B) and *lsa*(E) antibiotic resistance genes flanked by IS*1216*, effectively causing multidrug resistance. Collectively, we believe that our study identifies the underlying genes responsible for the pathogenesis of new GBS serotype III. Furthermore, our study emphasizes the need for alternative antibiotics for patients who are allergic to β-lactams and for those who are pregnant.

## INTRODUCTION

Streptococcus agalactiae (group B streptococcus [GBS]) is a Gram-positive commensal bacterium that colonizes the gastrointestinal, urinary, and genital tracts of healthy adults (1). Maternal GBS colonization is the leading cause of neonatal sepsis and meningitis and a significant cause of morbidity in immunocompromised adults, particularly the elderly (2, 3). Recent studies have reported that invasive GBS infections among the elderly were responsible for approximately 25 deaths/100,000 population in the United States in 2015 (4). The capsular polysaccharide (CPS), a major determinant in the categorization of GBS strains into 10 serotypes (Ia, Ib, and II to IX), is the most important virulence factor (5). However, several reports have described multilocus sequence typing (MLST) as another potential GBS classification system for describing the evolutionary relationships and epidemiology of prevalent sequence type (ST) isolates (6, 7). Using MLST, ST17 serotype III GBS strains, which currently account for the highest proportion of infant invasive disease, have been predicted as emerging highly virulent strains (8–11).

Penicillin G and ampicillin are the most widely used antibiotics for the prevention and treatment of GBS infections (12, 13); however, penicillin-nonsusceptible GBS isolates have been reported worldwide (14, 15). Meanwhile, erythromycin and clindamycin represent second-line antibiotics that are often recommended by the Centers for Disease Control and Prevention (CDC) and the American College of Obstetricians and Gynecologists (ACOG) for patients with allergies to β-lactam antibiotics (16).

In addition, owing to their synergistic activity, aminoglycosides are commonly prescribed in combination with β-lactam antibiotics for streptococcal endocarditis (17) and prosthetic joint infections (18). However, the increasing resistance rates reported in South Korea (51.8%), China (74.1%), and other countries against macrolide and aminoglycoside antibiotics pose a serious global clinical challenge (19, 20).

GBS can also develop resistance to multiple antimicrobial compounds due to the acquisition of resistance genes hosted on mobile genetic elements (MGEs) (21). MGEs comprise insertion sequences (ISs), transposons (Tns), plasmids, and bacteriophages (22), which can translocate within, or between, prokaryotic genomes. Many MGEs have a significant role in facilitating horizontal genetic transfer (HGT) and promoting the acquisition of antibiotic resistance genes (23). In fact, in GBS, in which lincosamide-resistant strains are rarely isolated, the emergence of clinical isolates resistant to lincosamides is increasing due to the acquisition of an *lnu*(B)-carrying fragment (24–29). For instance, the GBS isolate SGB76 harbors a 12,076-bp *lnu*(B)-carrying fragment along with other antibiotic resistance genes, including *aadE* (streptomycin resistance), *spw* (spectinomycin resistance), and *lsa*(E) (pleuromutilin, lincosamide, and streptogramin A resistance). These genes are located between two IS*1216* fragments that share a high degree of homology with the IS*1216* locus located in Staphylococcus aureus SA7037 and Enterococcus faecalis pEF418 (30, 31). Moreover, the GBS strain UCN70, isolated from a vaginal swab, was reported to be macrolide, lincosamide, and streptogramin A resistant (32). Indeed, the expansion of MGEs containing antibiotic resistance genes in GBS has been suggested to contribute to the emergence of adult infections in nonpregnant individuals (33–40).

Clinical GBS isolates encode serine-rich-repeat (SRR) glycoproteins (Srr1 or Srr2), important adhesion molecules that interact with human fibrinogen (41, 42). Based on their significant roles in the pathogenesis of GBS infections, Srr1 and Srr2 have been investigated as attractive novel vaccine candidates against GBS infections. However, we previously reported that 3.2% (*n* = 6) of 185 GBS clinical isolates, collected at the Korea University Hospital (Seoul, South Korea), did not carry *srr1* or *srr2*, and all belonged to serotype III (43). These findings prompted us to further investigate the correlation between non-*srr1/2* strains and GBS serotypes as well as the genomic characteristics associated with *srr1/2* deficiency in GBS clinical isolates. Therefore, in the current study, 41 non-*srr1/2* strains from 305 serotype III clinical isolates were analyzed using comparative whole-genome sequencing to investigate the relationship between their genetic structures and antibiotic resistance and virulence.

## RESULTS

### Epidemiological analysis of non-*srr1/2* GBS clinical isolates.

A total of 1,248 GBS clinical isolates were collected between 2013 and 2016 at the Korea University Guro Hospital (Seoul, South Korea). Capsular serotypes of the isolates were identified using a latex agglutination assay and multiplex PCR analysis, revealing 86 serotype Ia, 205 serotype Ib, 30 serotype II, 305 serotype III, 11 serotype IV, 124 serotype V, 99 serotype VI, 2 serotype VII, 185 serotype VIII, and 9 serotype IX isolates (Table 1). Additionally, 192 isolates were identified as nontypeable or multitypeable strains. Consistent with our previous report (43), serotype III (24.4%) represented the predominant GBS serotype. Interestingly, serotype VIII was also found to represent a major serotype isolated from the Korea University Guro Hospital, accounting for 14.8% of the clinical isolates and increasing from 7.0% in 2013 to 19.0% in 2016 during this study period.

**TABLE 1:** Capsular serotypes of GBS clinical isolates

Yr	No. (%) of isolates of serotype											
	Ia	Ib	II	III	IV	V	VI	VII	VIII	IX	NT^*a*^	Total
2013	16 (8.6)	32 (17.3)	6 (3.2)	41 (22.2)	2 (1.1)	20 (10.8)	16 (8.6)	0 (0.0)	13 (7.0)	1 (0.5)	38 (20.5)	185 (100)
2014	12 (4.2)	50 (17.4)	4 (1.4)	83 (28.9)	2 (0.7)	29 (10.1)	28 (9.8)	1 (0.3)	24 (8.4)	2 (0.7)	52 (18.1)	287 (100)
2015	29 (7.7)	63 (16.8)	11 (2.9)	97 (25.9)	3 (0.8)	31 (8.3)	28 (7.5)	1 (0.3)	72 (19.2)	0 (0.0)	40 (10.7)	375 (100)
2016	29 (7.2)	60 (15.0)	9 (2.2)	84 (20.9)	4 (1.0)	44 (11.0)	27 (6.7)	0 (0.0)	76 (19.0)	6 (1.5)	62 (15.5)	401 (100)

Total	86 (6.9)	205 (16.4)	30 (2.4)	305 (24.4)	11 (0.9)	124 (9.9)	99 (7.9)	2 (0.2)	185 (14.8)	9 (0.7)	192 (15.4)	1,248 (100)

a NT, nontypeable.

We had previously determined that all non-*srr1/2* strains were of serotype III (43), in the current study, we investigated the presence of the *srr1* and *srr2* genes in 305 serotype III isolates using a multiplex PCR assay. Among these isolates, 240 and 24 carried *srr1* and *srr2*, respectively (Table 2). Notably, the remaining 41 isolates were identified as non-*srr1/2* strains, accounting for 13.4% of serotype III and 3.3% of the total isolates. To further investigate the genetic characteristics of these strains, MLSTs and clonal complexes (CCs) of all 41 non-*srr1/2* isolates were analyzed. Among the 41 non-*srr1/2* isolates, 16 belonged to ST19, while the remaining 25 were of ST27 (*n* = 18), ST529 (*n* = 6), and ST171 (*n* = 1) (Table 3). eBURST analysis further revealed that all non-*srr1/2* isolates, with the exception of one isolate (NSP14-65 [ST171; CC17]), belonged to CC19 (44). Taken together, 41 isolates (13.4%) of the 305 serotype III isolates were identified as non-*srr1/2* strains, with the largest proportion belonging to CC19 (97.6%; *n* = 40).

**TABLE 2:** Prevalence of *srr* genotypes in serotype III isolates from 2013 to 2016

Genotype	No. of isolates from yr				Total no. (%) of isolates
	2013	2014	2015	2016	
*srr1*	29	66	78	67	240 (78.7)
*srr2*	0	4	10	10	24 (7.9)
Non-*srr1/2*	12	13	9	7	41 (13.4)

Total	41	83	97	84	305 (100.0)

**TABLE 3:** STs, clonal complexes, and MICs of 41 non-*srr1/2* isolates^*a*^

Isolate	ST	CC	MIC (μg/ml)							
			Aminoglycosides						Lincosamides	
			AMK	GEN	KAN	SPT	STR	TOB	CLI	LIN
S9968	19	19	128	512	512	512	64	512	4	32
S10039	19	19	128	512	512	512	64	512	64	128
S10124	19	19	128	512	512	512	256	512	128	256
S10171	19	19	128	512	512	512	256	512	4	32
NSP14-81	19	19	128	512	512	256	256	512	4	32
NSP14-151	19	19	128	512	512	32	64	512	128	256
NSP14-167	19	19	128	512	512	128	64	512	64	128
NSP15-560	19	19	128	512	512	512	64	512	64	128
NSP15-613	19	19	128	512	512	32	64	512	64	128
NSP16-136	19	19	128	512	512	32	32	256	64	128
GBS16-89	19	19	128	512	512	512	256	256	64	256
S10120	19	19	32	8	64	16	32	16	<0.5	<0.5
NSP14-66	19	19	16	4	32	32	16	8	<0.5	<0.5
NSP15-659	19	19	64	16	128	32	64	16	<0.5	<0.5
NSP16-31	19	19	64	8	64	32	64	16	<0.5	<0.5
GBS16-54	19	19	64	8	64	32	32	16	<0.5	<0.5
S9928	27	19	64	16	128	32	64	16	<0.5	<0.5
S9981	27	19	64	16	64	32	64	16	<0.5	<0.5
S10048	27	19	64	16	128	32	64	16	<0.5	<0.5
S10072	27	19	64	16	128	32	64	32	<0.5	<0.5
S10121	27	19	64	16	128	32	64	16	<0.5	<0.5
NSP14-76	27	19	64	16	64	32	64	16	<0.5	<0.5
NSP14-110	27	19	64	16	128	32	64	16	<0.5	<0.5
NSP14-149	27	19	64	8	64	32	64	16	<0.5	<0.5
NSP14-150	27	19	128	16	256	64	128	32	<0.5	<0.5
NSP14-161	27	19	32	8	64	32	32	8	<0.5	<0.5
NSP14-259	27	19	64	16	128	32	64	16	<0.5	<0.5
NSP15-73	27	19	32	8	64	32	64	16	<0.5	<0.5
NSP15-236	27	19	64	16	64	32	64	16	<0.5	<0.5
NSP15-531	27	19	64	16	128	32	64	16	<0.5	<0.5
NSP15-584	27	19	64	8	64	32	64	16	<0.5	<0.5
NSP15-635	27	19	64	16	128	32	64	16	<0.5	<0.5
NSP16-102	27	19	64	16	128	32	64	16	<0.5	<0.5
GBS16-40	27	19	32	8	64	16	32	8	<0.5	<0.5
S10003	529	19	64	8	64	32	32	16	<0.5	<0.5
S10161	529	19	64	16	64	32	32	16	<0.5	<0.5
NSP14-28	529	19	64	16	64	32	64	16	<0.5	<0.5
NSP14-87	529	19	64	16	128	32	64	16	<0.5	<0.5
NSP15-667	529	19	128	16	128	32	64	16	<0.5	<0.5
GBS16-17	529	19	64	8	64	32	64	16	<0.5	<0.5
NSP14-65	171	17	128	16	128	32	64	32	<0.5	<0.5

a Abbreviations: ST, sequence type; CC, clonal complex; AMK, amikacin; GEN, gentamicin; KAN, kanamycin; SPT, spectinomycin; STR, streptomycin; TOB, tobramycin; CLI, clindamycin; LIN, lincomycin.

### Phenotypic characteristics of non-*srr1/2* isolates.

**FIG 1:**
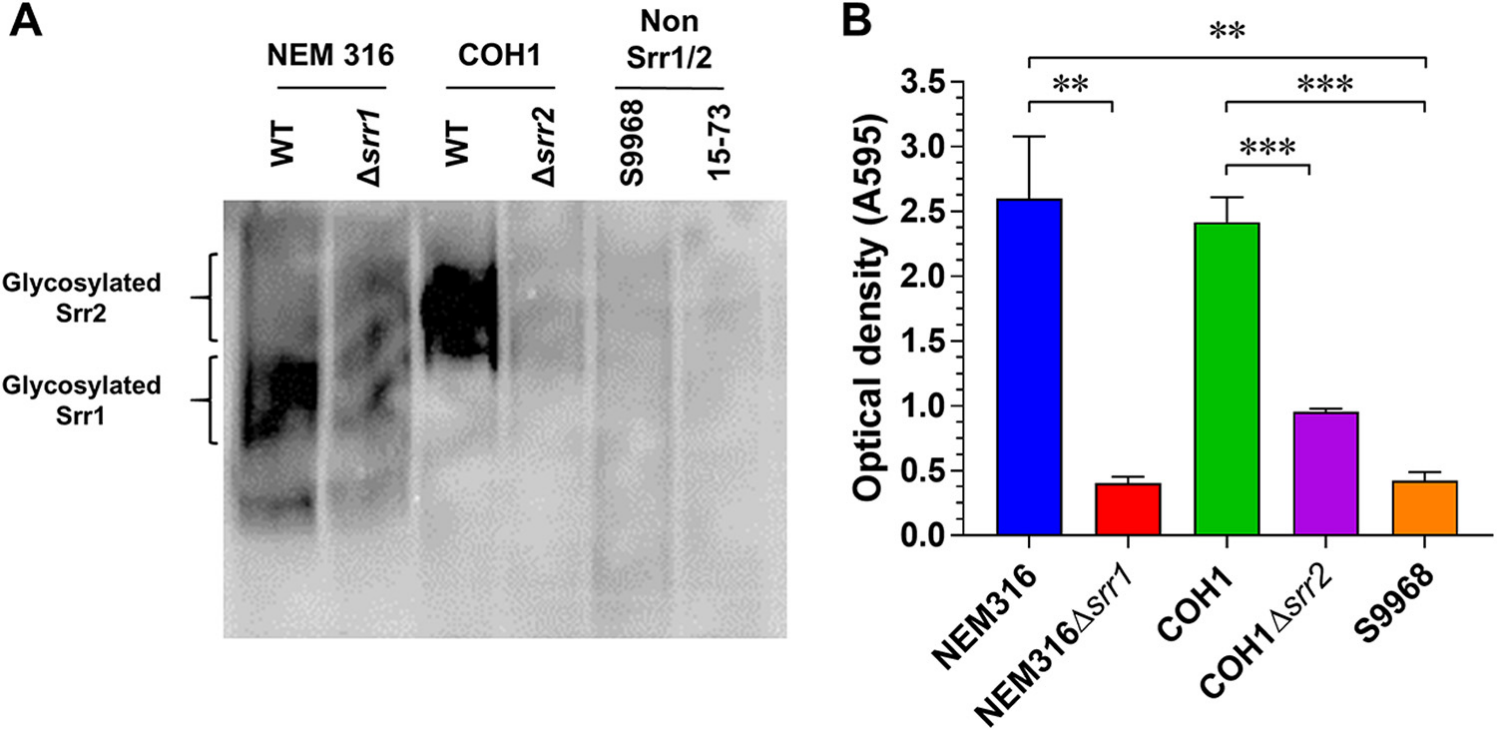
Reduced binding of non-*srr1/2* isolates to immobilized fibrinogen. (A) Expression of glycoproteins Srr1 and Srr2 on the cell surface. Cell wall proteins were isolated from Srr1-expressing GBS (NEM316) and its isogenic mutant (Δ*srr1*), Srr2-expressing GBS (COH1) and its isogenic mutant (Δ*srr2*), and non-*srr1/2* isolates S9968 and NSP15-73. Srr1/2 proteins separated by SDS-PAGE were detected with biotin-conjugated WGA, followed by incubation with HRP-conjugated streptavidin. (B) GBS binding to immobilized fibrinogen. Suspensions of GBS WT (NEM316, COH1, and S9968) and isogenic mutant (NEM316 Δ*srr1* and COH1 Δ*srr2*) strains were incubated in wells pretreated with human fibrinogen (0.1 μM), followed by detection with 0.1% crystal violet. Cell binding to immobilized fibrinogen was measured as the absorbance at 595 nm. Data are presented as the means ± standard deviations. **, *P* < 0.005; ***, *P* < 0.001.

Generally, GBS strains express one of the two SRR proteins Srr1 and Srr2, which are heavily glycosylated cell wall-anchoring proteins (45–47). Thus, the loss of *srr1* and *srr2* expression in S9968 (ST19) and NSP15-73 (ST27) was confirmed by lectin blot analysis. The results show that a strong glycosylated protein signal was detected in the NEM316 strain, which was lost in the isogenic *srr1* deletion mutant NEM316 (Δ*srr1*) (Fig. 1A). A strong glycosylated protein signal was also detected in the COH1 strain, which was lost upon *srr2* deletion (Δ*srr2*). In contrast, no glycosylated protein signals were observed in the non-*srr1/2* isolates S9968 and NSP15-73, suggesting the absence of Srr1/2 proteins in the non-*srr1/2* strain serotype III GBS isolates. This was confirmed following our examination of an additional five non-*srr1/2* strains, all of which lacked Srr1/2 protein expression (data not shown).

Considering the reported contribution of Srr1 or Srr2 to the colonization and virulence of GBS in the pathogenesis of meningitis and endocarditis via its interaction with human fibrinogen (48), we next sought to investigate the impact of *srr1/2* gene loss in non-*srr1/2* isolates on their fibrinogen binding activity. This activity was observed to be significantly reduced in the *srr1-* and *srr2-*deficient strains compared to that in their parental wild-type (WT) strains, NEM316 and COH1, respectively. Additionally, the binding of S9968 to fibrinogen was also markedly inhibited compared to that of *srr1-* and *srr2-*expressing WT strains (Fig. 1B). Moreover, we found that most of the 41 non-*srr1/2* isolates exhibited reduced fibrinogen binding, with the exception of NSP14-66 of ST19, NSP14-76 and NSP14-161 of ST27, and NSP14-28 of ST529 (see Fig. S1 in the supplemental material).

10.1128/mSphere.00543-21.1 FIG S1 Comparison of reduced fibrinogen binding affinities between *srr1* or *srr2* isogenic mutants and 41 non-*srr1/2* strains. Suspensions of WT GBS (NEM316 and COH1), isogenic mutant strains (NEM316 Δ*srr1* and COH1 Δ*srr2*), and 41 non-*srr1/2* strains were incubated in wells precoated with human fibrinogen (3 μg), followed by detection with 0.1% crystal violet. Cell binding to immobilized fibrinogen was measured as the OD_595_. Each dot represents the mean from triple experiments with one GBS isolate. Download FIG S1, TIF file, 1.2 MB. Copyright © 2021 Zhi et al. 2021 Zhi et al. https://creativecommons.org/licenses/by/4.0/ This content is distributed under the terms of the Creative Commons Attribution 4.0 International license.

The antibiotic susceptibility of non-*srr1/2* isolates (*n* = 41) was then evaluated. All of these isolates were found to exhibit high levels of resistance to the six tested aminoglycoside antibiotics (amikacin [AMK], gentamicin [GEN], kanamycin [KAN], spectinomycin [SPT], streptomycin [STR], and tobramycin [TOB]), with MICs ranging from 8 to over 512 μg/ml (Table 3). Among them, 11 isolates showed extremely high-level resistance to clindamycin and lincomycin (LIN), with MICs ranging from 4 to >256 μg/ml. Moreover, these 11 isolates, all of which belonged to ST19, exhibited higher resistance to gentamicin, kanamycin, and tobramycin (MIC values of over 256 μg/ml) than the remaining 30 isolates.

### Whole-genome and comparative genomic analyses of non-*srr1/2* strains.

To confirm the genetic organization of non-*srr1/2* isolates of GBS serotype III, whole genome analysis was performed using hybrid sequencing with the Pac-Bio and Illumina MiSeq platforms. The whole genomes of S9968 (ST19), isolated from the urine of a 48-year-old female patient with chronic renal failure and urinary tract infections, and NSP15-73 (ST27), isolated from the urine of a 50-year-old female patient with chronic renal failure and urinary tract infections, were analyzed (Table S1) to determine whether any genetic characteristics were unique to non-*srr1/2* isolates. The circular chromosomes of S9968 and NSP15-73 were found to be 2,201,113 and 2,302,798 bp long, respectively, which were slightly longer than those of other sequenced serotype III strains, such as COH1 (CC17; 2,065,074 bp) and H002 (CC19; 2,147,420 bp). Additionally, the percent GC contents of S9968 (36.0%) and NSP15-73 (35.8%) were similar to those of COH1 (35.5%) and H002 (35.7%). Among the total 2,251 (S9968) and 2,348 (NSP15-73) genes, 2,149 and 2,246 were protein encoding, respectively. Additionally, the numbers of rRNAs and tRNAs in both strains were 21 and 80, respectively (49, 50). In the NSP15-73 strain, all accessory Sec2 system genes were absent, and no other gene substitutions or mutations were detected (Fig. S2). Considering the clinical importance of clindamycin and lincomycin in treating GBS infection in patients with allergies to penicillin, we next focused on the characteristics of non-*srr1/2* isolates of GBS serotype III with aminoglycoside and lincosamide resistance patterns.

10.1128/mSphere.00543-21.2 FIG S2 Schematic representation of the deletion locus flanked by two IS*1216* transposases in NSP15-73. The genetic locus flanked by two IS*1216* transposases in the site 4 cluster of the NSP15-73 strain was compared with the corresponding regions identified in GBS strain NEM316. Download FIG S2, TIF file, 1.1 MB. Copyright © 2021 Zhi et al. 2021 Zhi et al. https://creativecommons.org/licenses/by/4.0/ This content is distributed under the terms of the Creative Commons Attribution 4.0 International license.

10.1128/mSphere.00543-21.4 TABLE S1 Strain information for 41 non-*srr1/2* clinical isolates. Download Table S1, DOCX file, 0.02 MB. Copyright © 2021 Zhi et al. 2021 Zhi et al. https://creativecommons.org/licenses/by/4.0/ This content is distributed under the terms of the Creative Commons Attribution 4.0 International license.

**FIG 2:**
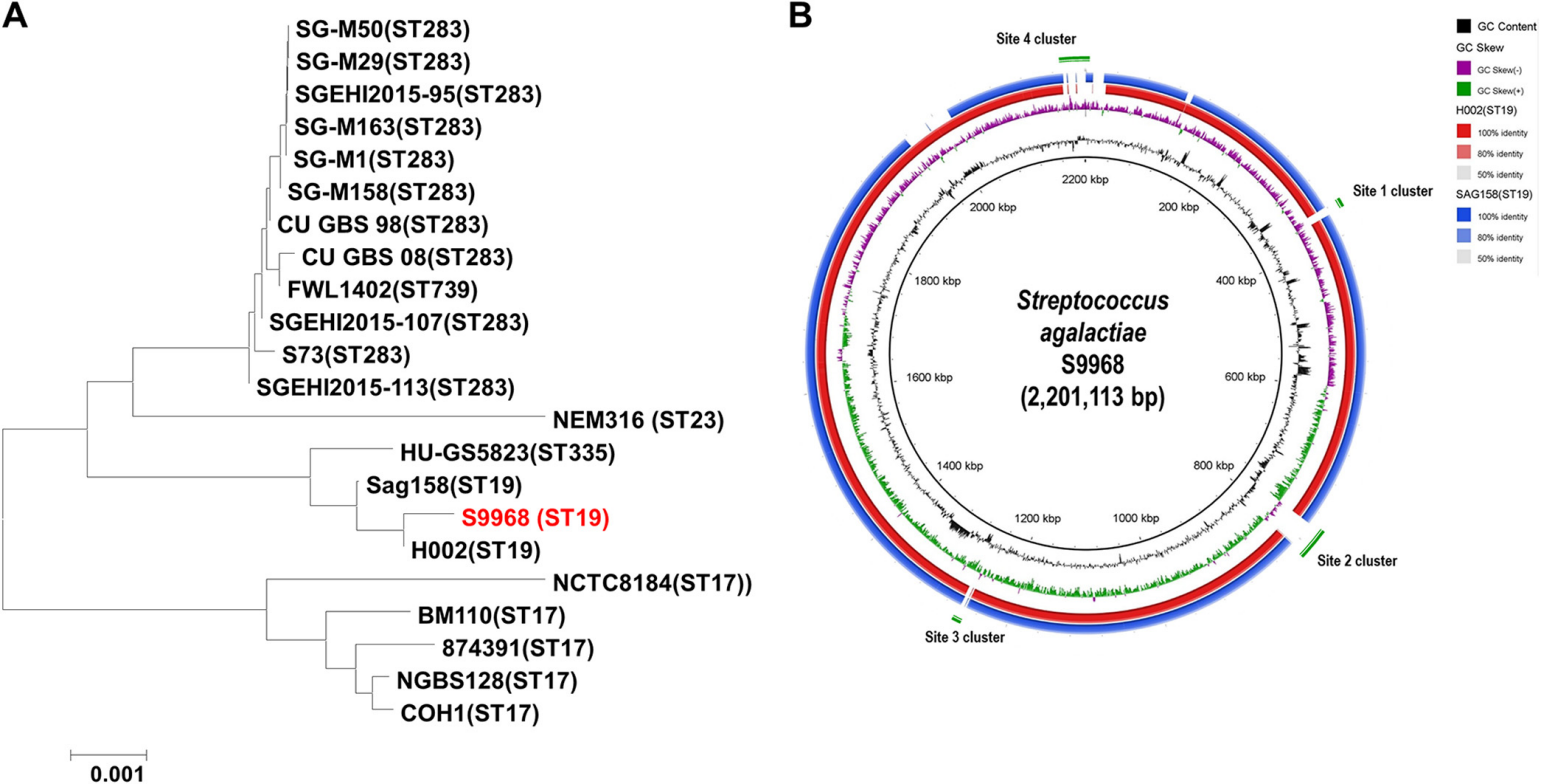
Genome comparison of GBS S9968 and other serotype III isolates. (A) Phylogenetic tree analysis of the S9968 strain based on ANI values obtained from whole genome sequences. The genome sequences were retrieved from the GenBank database, and ANI values were estimated using JSpecies. The phylogenetic tree was constructed using MEGA 6.0. (B) Comparative genome visualization map of S9968 with two related genome isolates, Sag158 (GenBank accession number CP019979) and H002 (accession number CP011329). GC contents and GC skew are represented on the distance scale in the inner map. Orthologous genes found in H002 (red) and Sag158 (blue) are displayed in the outer map. The double line (green) in the map indicates the unique ORFs found in S9968.

Average nucleotide identity (ANI) is considered the most relevant comparative parameter used for bacterial species delineation (51). The phylogenetic analysis of S9968 compared to 21 serotype III reference strains showed that S9968 had ANI values of >99% with the other reference serotype III strains, with the exception of CNCTC8184 (98.4%) and NEM316 (98.7%), and was found to be the most closely related to H002 (99.93%) and Sag158 (99.76%), both of which belong to ST19 (Fig. 2A).

BLAST Ring Image Generator (BRIG) software was then used to create the circular structure of the S9968 genome, which was then compared with those of H002 (STIII; ST19) and Sag158 (STIII; ST19) (52). Four large novel cluster sites unique to S9968 were found (Fig. 2B). Site 1 contained 15 genes, including 3 transposons and 1 integrase. Site 2 comprised 36 genes, including that encoding the conjugal transfer protein TraG as well as several conjugation-related genes encoding a conjugative transposon, a relaxase, a relaxosome, and a DNA primase. In addition, three LPxTG cell wall-anchoring proteins were encoded in site 2. Meanwhile, site 3 and site 4 were the contigs flanked by the IS*6*-like element IS*1216*. Site 3 harbored 19 genes, including the *spw* gene belonging to the ANT (9) family of aminoglycoside nucleotidyltransferases, which confers resistance to spectinomycin. The site 4 cluster, found in the *srr1* gene locus, comprised 29 genes located upstream of the accessory *sec2* system and spanned 11,886 bp (Table S2). In addition, PCR and sequencing analyses (Fig. S3) revealed that among the 41 non-*srr1/2* isolates, only those with both aminoglycoside and lincosamide resistance (*n* = 11) harbored the *lnu*(B)-carrying fragment (Fig. 2B).

10.1128/mSphere.00543-21.3 FIG S3 Analysis of the IS*1216* diagnostic PCR region of the 41 non-*srr1/2* strains’ site 4 cluster by using agarose gel electrophoresis (0.75%) for PCR. The PCR conditions were as follows: 1 cycle at 95°C for 5 min; 30 cycles of 95°C for 20 s, 56.2°C for 20 s, and 72°C for 3.5 min; and 1 cycle of 72°C for 5 min. The predicted PCR product size was 3,317 bp. Download FIG S3, TIF file, 2.2 MB. Copyright © 2021 Zhi et al. 2021 Zhi et al. https://creativecommons.org/licenses/by/4.0/ This content is distributed under the terms of the Creative Commons Attribution 4.0 International license.

10.1128/mSphere.00543-21.5 TABLE S2 Genes in site 1, 2, 3, and 4 clusters of S9968. Download Table S2, DOCX file, 0.03 MB. Copyright © 2021 Zhi et al. 2021 Zhi et al. https://creativecommons.org/licenses/by/4.0/ This content is distributed under the terms of the Creative Commons Attribution 4.0 International license.

### Genetic environment of IS*1216* contigs in S9968.

**FIG 3:**
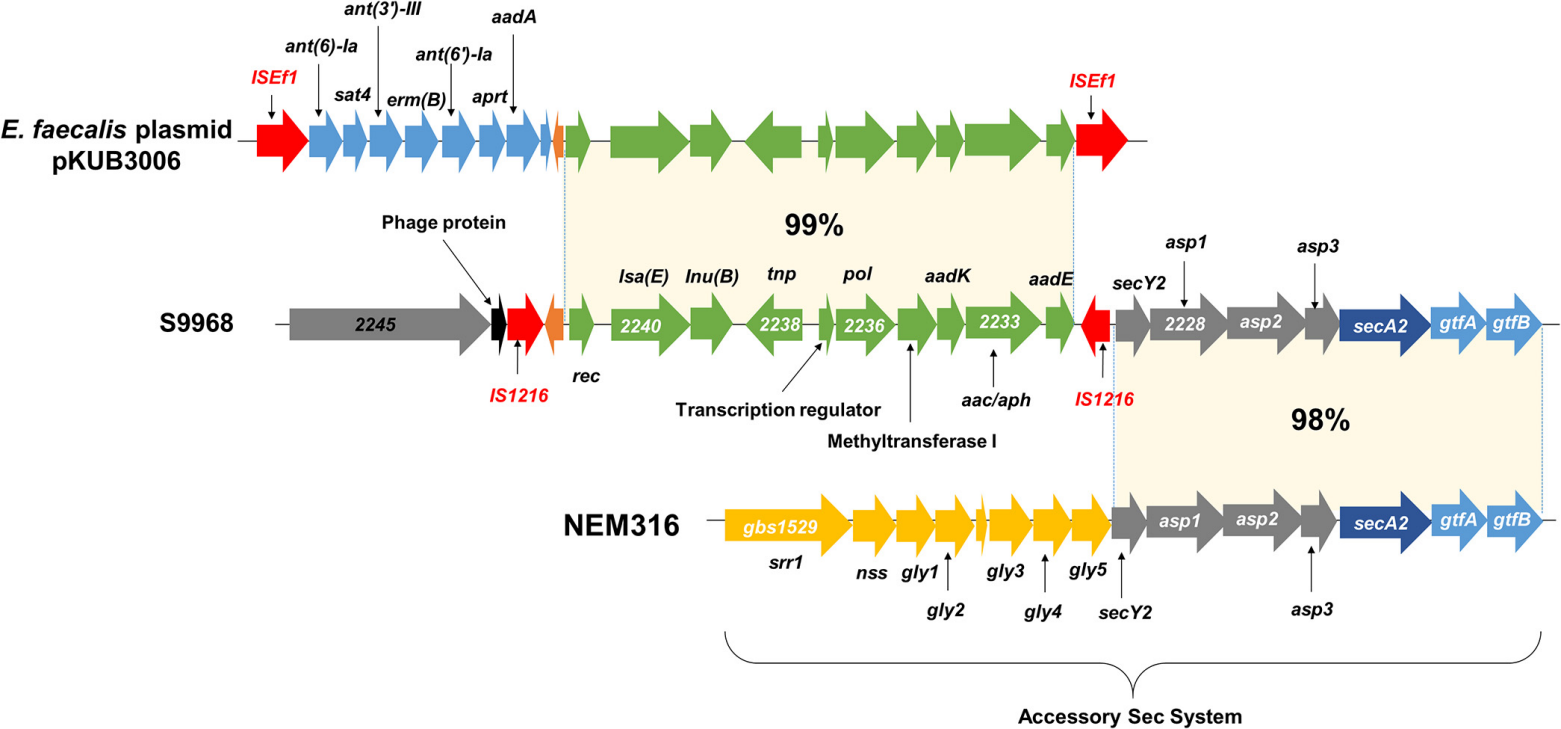
Schematic representation of the antibiotic resistance locus flanked by two IS*1216* transposases in GBS S9968. The genetic locus flanked by two IS*1216* transposases in the site 4 cluster of the S9968 strain was compared with the corresponding regions identified in GBS strains H002 and NEM316 and E. faecalis pKUB3006.

Comparative genomic analysis showed that the site 4 cluster of S9968 comprised 12 open reading frames (ORFs), which contained *lsa*(E) for pleuromutilin-lincosamide-streptogramin A resistance, *lnu*(B) for lincosamide resistance, *aadK* and *aadE* for streptomycin resistance, and *aac*-*aph* for gentamicin-kanamycin-tobramycin resistance (Fig. 3). Unlike the previously reported *lnu*(B)-carrying fragment in GBS (28, 31), which carries *aadE*, *spw*, and *lsa*(E), the *lnu*(B)-carrying fragment of S9968 contains additional aminoglycoside resistance genes, *aadK* and *aac-aph* (31). In addition, the *spw* gene, which was absent from the site 4 cluster, was located in the site 3 cluster.

Basic Local Alignment Search Tool Nucleotide (BLASTN) analysis further indicated that antibiotic resistance genes (12 ORFs) in the site 4 cluster showed high similarity to those of several multidrug-resistant bacteria, including Staphylococcus and *Enterococcus*. Among them, enterococcal plasmid pKUB3007-1 carrying antibiotic resistance genes, flanked by two IS*Ef1* elements, shared the highest nucleotide homology with the *lnu*(B)-carrying fragment of S9968 (99% maximum identity and 99.89% query coverage of the total nucleotide sequence) (53). However, it exhibited low homology with previously reported GBS *lnu*(B)*-lsa*(E) transposons (31).

### Virulence of non-*srr1/2* isolates.

**FIG 4:**
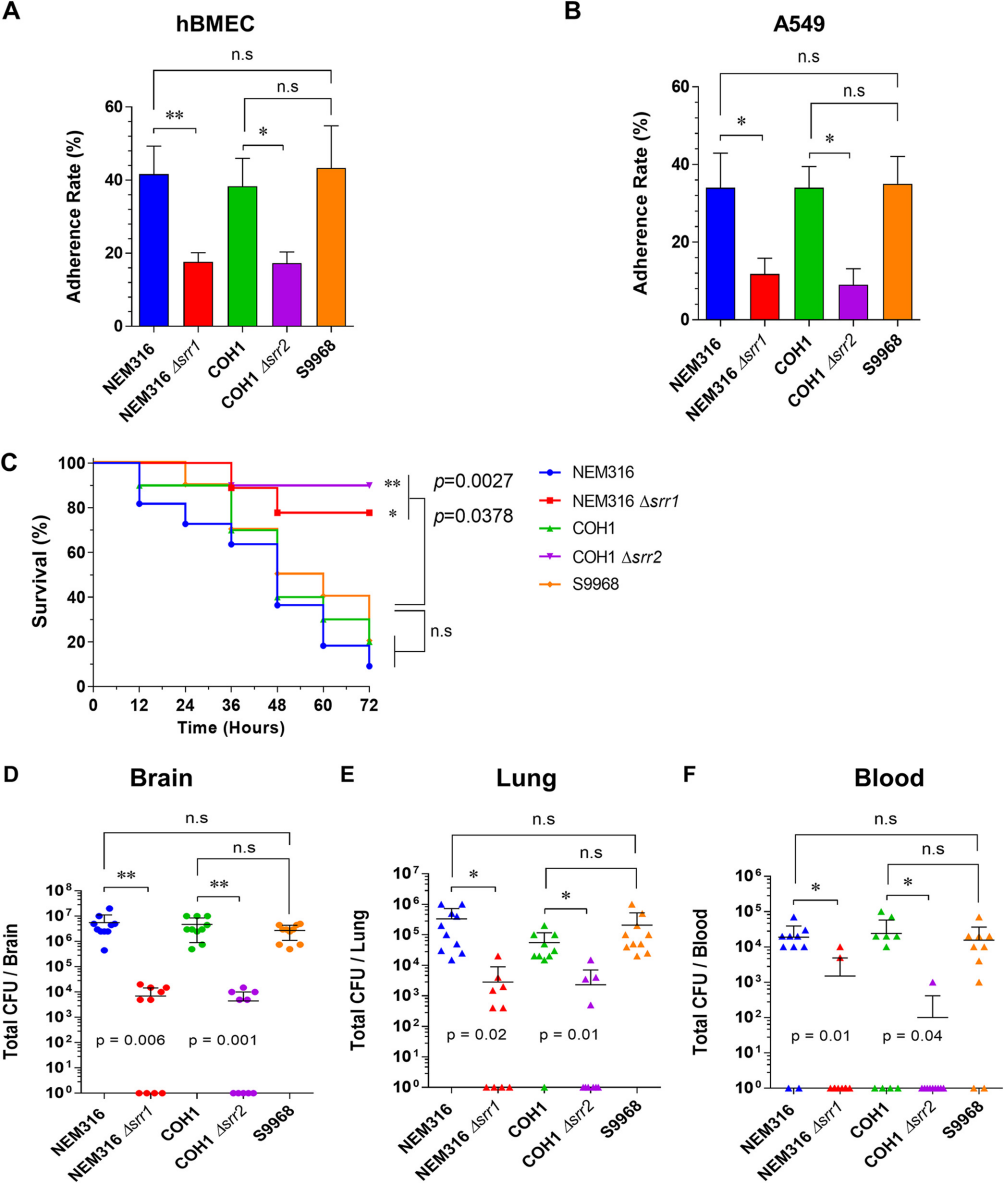
Comparison of adherence rates and virulences of GBS strains. (A and B) Adherence of GBS strains, NEM316 and its isogenic mutant (Δ*srr1*), COH1 and its isogenic mutant (Δ*srr2*), and S9968, to hBMECs (A) and A549 cells (B). Data are presented as the means ± standard deviations. (C) Kaplan-Meier survival curves. CD-1 male mice were injected i.v. with GBS strain NEM316 or its isogenic mutant (Δ*srr1*), COH1 or its isogenic mutant (Δ*srr2*), or S9968 (*n* = 10 per group). Mouse survival was monitored for 72 h. (D to F) Bacterial loads in mouse organs. At the end of the experiment, mice were euthanized, and bacterial loads in the brain (D), lung (E), and blood (F) were assessed. *, *P* < 0.05; **, *P* < 0.01; n.s, not significant.

Srr1 and Srr2 serve as important adhesins involved in GBS invasion via the blood-brain barrier (54). In fact, deletion of *srr1* or *srr2* in GBS resulted in significantly lower levels of invasiveness in human brain microvascular endothelial cells (hBMECs) than those of WT GBS strains (41). Thus, to determine whether non-*srr1/2* clinical isolates exhibit impaired binding to endothelial and epithelial cells, we examined if the adherence of S9968 to hBMECs and A549 cells differed compared with that of Srr1- and Srr2-expressing serotype III reference strains (NEM316 and COH1, respectively). The mutant strains deficient in *srr1* or *srr2* (NEM316 Δ*srr1* or COH1 Δ*srr2*) showed significantly diminished adherence to both hBMECs and A549 cells compared to their parental WT strains (NEM316 or COH1) (Fig. 4A and B). However, despite the defect in both the *srr1* and *srr2* genes, the capacity for adherence of the S9968 strain to hBMECs and A549 cells did not differ significantly from that of NEM316 and COH1.

Finally, the virulences of S9968 and Srr1- or Srr2-expressing serotype III strains (NEM316 or COH1, respectively) were compared in a mouse model of GBS meningitis. CD-1 mice (*n* = 10 per group) were infected intravenously (i.v.) with either NEM316, COH1, or S9968, and their survival was monitored over 72 h (Fig. 4C). The results showed that 80% of the mice infected with COH1 or S9968 and 90% of the mice infected with NEM316 died within 72 h, with no significant differences among the three groups. In contrast, *srr1-* or *srr2-*deficient isogenic mutant strains exhibited significantly lower virulence than their parental strains (NEM316 and COH1) or S9968. Mice were euthanized, and brain, blood, and lung tissues were collected to enumerate the bacterial loads. As shown in Fig. 4D to F, mice infected with *srr1-* or *srr2-*deficient isogenic mutants exhibited significantly lower bacterial loads in the brain, lung, and blood than those infected with the parental strains (NEM316 and COH1) or S9968.

Collectively, these findings suggest that despite the defect in Srr1 or Srr2 expression and the failure to bind fibrinogen, non-*srr1/2* clinical isolates exhibited capacities for binding to brain cells and virulence comparable to those of Srr1- or Srr2-expressing isolates.

## DISCUSSION

The geographic distribution of GBS serotypes varies across countries and over time. This study comprised capsular serotyping of 1,248 GBS clinical isolates collected over 4 years at the Korea University Guro Hospital, located in the southwestern region of Seoul, South Korea, which is inhabited by a diverse Asian population. Consistent with our previous report, serotype III was the most predominant serotype detected among the 10 capsular serotypes. Previously, serotype III was reported to account for 57.7% of invasive infections in neonates, 84.3% of infections in geriatric adults (55), and 21 to 28% of infections in pregnant women (56).

Although the cause of this increased invasiveness associated with serotype III GBS compared to other serotypes has not been fully elucidated, many clinical studies suggest that the lower level of production of CPS-specific antibodies during GBS infections with serotype III may be partially responsible. For example, neonatal infections with serotype III are likely associated with poor maternal transfer of serotype III CPS-specific IgG through the placenta, compared to other serotypes. Resistance to various antibiotics caused by the overprescription of antibiotics is considered another factor contributing to the climbing number of GBS infections in both neonates and adults (57). Among the 1,248 clinical isolates examined in the current study, we observed a pattern of high lincosamide resistance in serotype III. Further genomic analysis revealed that some serotype III isolates acquired more than two IS*1216* elements encoded by several antibiotic resistance genes. The first IS*1216* element, located in the site 3 cluster, carried only aminoglycoside resistance genes, while the second IS*1216* element, located in the site 4 locus, carried both aminoglycoside and lincosamide resistance genes.

IS*1216* is highly associated with several resistance genes in *Enterococcus* (58), Staphylococcus (58), and Streptococcus (59) species. ISFinder (https://isfinder.biotoul.fr) analysis showed that the IS*1216*-flanked genes identified in this study (designated IS*1216*-G1 and -G2 [808 bp]) shared 99% homology with IS*1216E* (IS*Efa14*; IS*S1Y*) from Enterococcus faecium CH19 (GenBank accession number U49512) (60). Furthermore, this IS*1216* element comprised 226-amino-acid (aa) transposases (Tnps) with a DDE motif for strand cleavage and rejoining as well as 24 bp of a left inverted repeat (LIR) (5′-GGT TCT GTT GCA AAG TTT TAA ATC TAC TAT CAA ATA AGG TAG AAT AG-3′) and a right inverted repeat (RIR) (5′-GGT TCT GTT GCA AAG TTT TAA ATA AAG AAT AAA ATC CTT ACG GTA TCT AT-3′). Although IS*1216* transposases carrying lincosamide resistance genes have been previously reported in GBS (28), this is the first study to report the presence of IS*1216* carrying two types of antibiotic resistance genes that are highly homologous to a portion of the E. faecalis plasmid pKUB3007-1.

IS*1216* found in the site 4 cluster carried multiresistance genes, including *lsa*(E) and *lnu*(B) for lincosamide resistance and *aadK*, *aacA*-*aphD*, and *aadE* for aminoglycoside resistance. Although lincosamides such as clindamycin are recommended alternatives for pregnant women and patients who are allergic to β-lactams (16), resistance to this class of drugs has been reported in GBS clinical isolates since the 1990s, which has been shown to be caused primarily by the *lnu*(B) gene alone or together with *lsa* (31, 61). Similarly, combination treatment with a β-lactam and an aminoglycoside is commonly administered for at least the first 2 weeks of infective endocarditis (62) and prosthetic joint infection (63) treatment; however, the emergence of resistant strains suggests that these agents should be used with caution.

Transposable elements may alter gene expression via insertion within the coding region or the promoter region to enhance or reduce host virulence. For instance, IS*Efa4* of E. faecalis disrupts the *ddl* gene encoding the housekeeping d-alanine-d-alanine ligase, the absence of which results in reduced glycopeptide resistance in E. faecalis (64). In the current study, non-*srr1/2* clinical isolates showed the transposition of IS*1216* in the accessory Sec system exporting LPxTG-anchoring glycoproteins (Srr1 or Srr2). Due to the insertion of IS*1216*, *srr1* was completely deleted in 11 non-*srr1/2* isolates.

The expression of Srr1 or Srr2 promotes GBS attachment to human brain endothelial cells via interaction with the fibrinogen Aα chain (48). Previous *in vivo* studies with targeted knockdown of the *srr1* or *srr2* gene have shown that *srr1* or *srr2* deficiency in GBS results in reduced virulence and colonization compared to those of parental WT strains (41). Similarly, the S9968 strain exhibited significantly lower binding of immobilized human fibrinogen than *srr1*- or *srr2*-expressing strains (NEM316 or COH1, respectively); however, its binding of endothelial and epithelial cells was comparable to that of the Srr1- and Srr2-expressing strains. Additionally, the virulence of S9968 did not differ significantly from that of Srr1- or Srr2-expressing strains in a murine model of invasive GBS infection. Comparative analysis of its genome with that of NEM316 or its closely related strain H002 revealed additional LPxTG motif-containing proteins, antigen I//II and BspA, in the site 2 gene cluster of S9968. Although the involvement of these proteins in GBS pathogenesis was not investigated in the current study, previous studies have reported that BspA and BspC are critical adhesins in GBS, which interact with the gp340 protein on human epithelial cells and the host cytoskeleton component vimentin, thereby contributing to GBS meningitis pathogenesis (65, 66). Thus, it is hypothesized that the loss of Srr1 or Srr2 in S9968, and the resulting decrease in fibrinogen binding, might be compensated by the acquisition of other cell wall-anchoring proteins, such as the BspA isotype. In fact, four isolates (NSP14-66, NSP14-76, NSP14-161, and NSP14-28) showed fibrinogen binding activity similar to or higher than that of Srr1- or Srr2-expressing serotype III strains (NEM316 or COH1). Thus, further investigation is required to define the pathogenesis of non-*srr1/2* isolates as well as effective therapeutic strategies.

The results presented in this study describe IS*1216* carrying lincosamide and aminoglycoside resistance genes in GBS, which might be acquired from other common multidrug-resistant bacteria such as *Enterococcus* spp. or Staphylococcus spp. Since all GBS clinical isolates have been reported to express either Srr1 or Srr2 protein on their cell wall, they have been considered potential vaccine candidates (43). However, this study reports the presence of GBS clinical isolates that do no express Srr1/2 following the insertion of IS*1216*. Thus, new strategies to develop a versatile GBS protein-based vaccine are warranted. In addition, considering the high level of resistance to lincosamide in GBS, alternative antibiotic agents should be considered for patients who are allergic to β-lactam antibiotics. Collectively, the results of this study provide important genomic evidence related to antibiotic resistance in clinical GBS isolates, which could facilitate the development of more effective treatment options.

## MATERIALS AND METHODS

### Chemical reagents.

All chemicals used in this study were purchased from Sigma-Aldrich (St. Louis, MO, USA) unless otherwise indicated.

### Bacterial strains.

The study was approved by the Korea University Guro Hospital (KUGH IRB number 2016GR0265). All clinical isolates (*n* = 1,248) were collected between 2013 and 2016 at the Korea University Guro Hospital (Seoul, South Korea), and all non-*srr1/2* clinical isolates used in this study are listed in Table S1 in the supplemental material. GBS reference strains and their Srr-deficient isogenic mutants, NEM316, NEM316 Δ*srr1*, COH1, and COH1 Δ*srr2*, were kindly provided by Paul Sullam (University of California, San Francisco, San Francisco, CA, USA).

### Capsular and genetic analyses of GBS isolates.

Capsular serotyping was conducted using the Strep-B-Latex kit (Statens Serum Institute, Copenhagen, Denmark) and a multiplex PCR assay (Table S3) as described previously (67, 68). The gene cluster flanked by two IS*1216* elements was detected by PCR specific for the targeted region from *pol* to *aadE* (Fig. 3 and Fig. S3). The *srr1* and *srr2* genes were also detected by PCR using specific primers. All primers are listed in Table S4.

10.1128/mSphere.00543-21.6 TABLE S3 Primers for multiplex PCR serotyping. Download Table S3, DOCX file, 0.02 MB. Copyright © 2021 Zhi et al. 2021 Zhi et al. https://creativecommons.org/licenses/by/4.0/ This content is distributed under the terms of the Creative Commons Attribution 4.0 International license.

10.1128/mSphere.00543-21.7 TABLE S4 Primers used for the PCR detection of *srr1/2* genes and IS*1216*. Download Table S4, DOCX file, 0.02 MB. Copyright © 2021 Zhi et al. 2021 Zhi et al. https://creativecommons.org/licenses/by/4.0/ This content is distributed under the terms of the Creative Commons Attribution 4.0 International license.

### Multilocus sequencing typing.

GBS multilocus sequence typing (MLST) was performed as described previously (69). Seven housekeeping genes (*adhP*, *pheS*, *atr*, *glnA*, *sdhA*, *glcK*, and *tkt*) were amplified and sequenced using MLST primer sets (Table S5), and PCR cycles were as follows: 1 cycle at 95°C for 5 min; 30 cycles of 95°C for 20 s, 56.2°C for 20 s, and 72°C for 1.5 min; and 1 cycle of 72°C for 5 min. The identified alleles were submitted to the S. agalactiae MLST database (http://pubmlst.org/sagalactiae/) for the assignment of sequence types (STs). In addition, each ST sharing six of the seven MLST loci with another ST in the group was clustered into a CC as described previously (70).

10.1128/mSphere.00543-21.8 TABLE S5 Primers for multilocus sequencing typing. Download Table S5, DOCX file, 0.02 MB. Copyright © 2021 Zhi et al. 2021 Zhi et al. https://creativecommons.org/licenses/by/4.0/ This content is distributed under the terms of the Creative Commons Attribution 4.0 International license.

### Antimicrobial susceptibility testing.

The broth microdilution test was used to determine the antimicrobial susceptibilities of GBS non-*srr1/2* clinical isolates to amikacin (AMK), gentamicin (GEN), kanamycin (KAN), spectinomycin (SPT), streptomycin (STR), tobramycin (TOB), clindamycin (CLI), and lincomycin (LIN) as described previously (71, 72). Briefly, GBS was inoculated in Todd-Hewitt broth supplemented with yeast extract (THY) and incubated overnight at 37°C. The culture of GBS grown overnight was diluted to achieve turbidity of an optical density at 600 nm (OD_600_) of 0.05 to 0.06, and 100 μl of the GBS suspension was then added to each well of a round-bottom 96-well plate (SPL, Pocheon, South Korea) with 2-fold serially diluted antibiotics. The breakpoints according to the criteria of the European Committee on Antimicrobial Susceptibility Testing were used to interpret the final MICs (73).

### Detection of Srr1 and Srr2 proteins using the lectin blot assay.

GBS cell wall extracts, prepared as described previously (41), were separated using 8 to 12% Bis-Tris gels (Invitrogen) and transferred to nitrocellulose membranes. The membranes were then incubated with biotin-conjugated wheat germ agglutinin (WGA; Vector Labs, Burlingame, CA, USA), followed by incubation with horseradish peroxidase (HRP)-conjugated streptavidin (0.2 μg/ml; Sigma-Aldrich). Signals were visualized using a chemiluminescent Western blotting substrate (Thermo Scientific, Waltham, MA, USA), and data were acquired using the Bio-Rad ChemiDoc Touch imaging system (Bio-Rad Laboratories, Hercules, CA, USA).

### Binding of GBS to immobilized fibrinogen.

Purified human fibrinogen (3 μg; Sigma-Aldrich) in phosphate-buffered saline (PBS) was immobilized in 96-well microtiter plates (SPL), and the wells were then blocked with 350 μl of a casein-based blocking solution (Sigma-Aldrich) for 1 h at room temperature. The plates were then incubated with 100 μl of the GBS suspension (10^10^ CFU/ml) for 30 min at 37°C and washed to remove unbound bacteria. Wells were stained with crystal violet (0.5% [vol/vol]; Sigma-Aldrich) as described previously (48), and the absorbance was measured at 595 nm using a spectrometer (Epoch 2; BioTek, Winooski, VT, USA).

### Whole-genome sequencing, genome assembly, and annotation.

The GBS clinical isolate S9968 (STIII; non-*srr1/2*; MLST ST19) was sequenced using PacBio single-molecule sequencers (Pacific Biosciences, Menlo Park, CA, USA), performed by Macrogen Inc. (Seoul, South Korea). *De novo* assembly was implemented using Hierarchical Genome Assembly Process version 3 (HGAP3). The genome annotations were performed using the PROKKA pipeline (v1.13), and gene functions were identified using eggNOG (74, 75). The GenBank accession number for the genomic sequence of the GBS strain S9968 is SAMN15246708. Comparative genomic analysis was performed by analyzing ANI for nucleotide-level comparisons with 21 serotype III strains retrieved from the NCBI GenBank database (Table S6) (51). The phylogenetic tree of 22 GBS strains, 21 serotype III reference strains, and the S9968 clinical isolate was constructed using MEGA 6.0 based on the ANI values (52). Genome-wide visualization of coding sequence identities between S9968 and other genomes of serotype III strains was performed using BRIG.

10.1128/mSphere.00543-21.9 TABLE S6 GBS accession numbers used for genome comparison. Download Table S6, DOCX file, 0.02 MB. Copyright © 2021 Zhi et al. 2021 Zhi et al. https://creativecommons.org/licenses/by/4.0/ This content is distributed under the terms of the Creative Commons Attribution 4.0 International license.

### GBS adherence assay.

Human brain microvascular endothelial cells (hBMECs; Lonza, Basel, Switzerland) and human alveolar epithelial cells (A549; ATCC, Manassas, VA, USA) were cultured in 24-well cell culture plates. When the cells reached approximately 90% confluence, they were washed before bacterial infection. GBS was then added to the cells at a multiplicity of infection (MOI) of 10 for 1 h. The monolayer was washed six times with PBS, followed by lysis with 0.05% trypsin-EDTA and 0.25% Triton X-100 after a 30-min incubation at 37°C (76). The lysates were serially diluted and plated onto blood agar plates to enumerate the bacteria. The adherent GBS bacteria were then calculated as follows: (recovered CFU/original CFU) × 100%.

### Mouse model of meningitis.

Animal experiments conducted in this study were approved by the Committee on the Use and Care of Animals at the Korea Energy Research Institute (KAERI-IACUC-2019-008 and KAERI-IACUC-2021-002) and were performed according to accepted veterinary standards. A murine model of hematogenous GBS meningitis was described previously (41). Groups (*n* = 10 per group) of outbred 6-week-old male CD-1 mice (OrientBio, Suwon, South Korea) were injected via the tail vein with 100 μl of PBS containing 5 × 10^7^ CFU of GBS (NEM316, NEM316 Δ*srr1*, COH1, COH1 Δ*srr2*, or S9968). Mouse survival was monitored for 72 h. At the experimental endpoint, the remaining mice were euthanized, and blood and brain tissues were collected. The tissues were homogenized, and the blood as well as the brain and lung homogenates were plated on blood agar for the enumeration of bacterial CFU.

### Statistics analysis.

Differences in the bacterial strains in the *in vitro* assays were evaluated using unpaired two-tailed Student’s *t* test. The survival of mice was determined using Kaplan-Meier survival analysis; data were representative of results from three independent experiments and expressed as the means ± standard deviations using GraphPad Prism version 6.0 (GraphPad Software Inc., La Jolla, CA, USA). A *P* value of <0.05 was considered statistically significant.
