# Supplementary figures and images for: Molecular Characteristics of IS1216 Carrying Multidrug Resistance Gene Cluster in Serotype III/Sequence Type 19 Group B Streptococcus
Source: mSphere. 2021 Jul 28;6(4):e00543-21. doi: 10.1128/mSphere.00543-21 (PMC8386385; doi:10.1128/mSphere.00543-21)

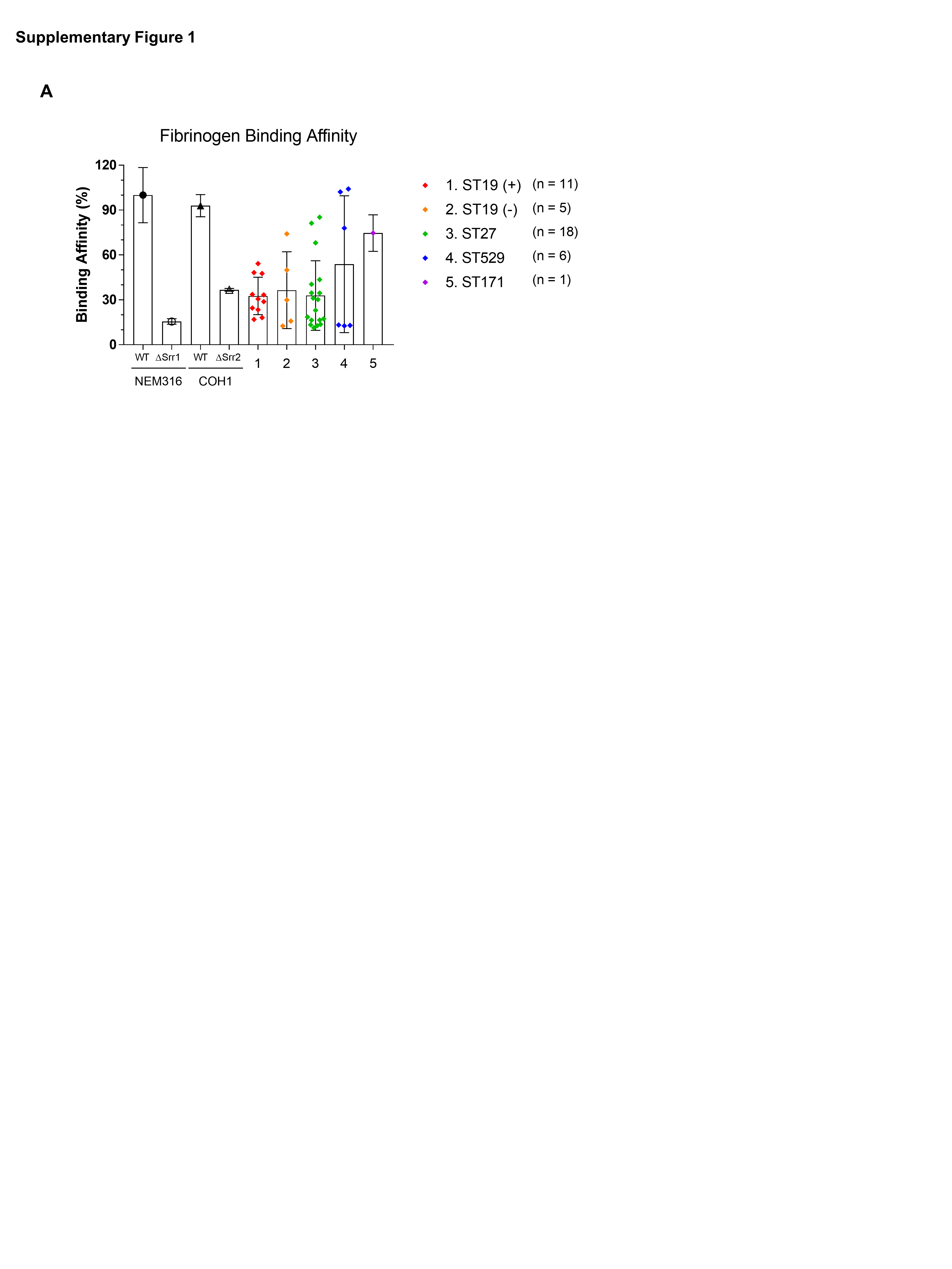

Supplement: FIG S1 [file msphere.00543-21-sf001.tif]

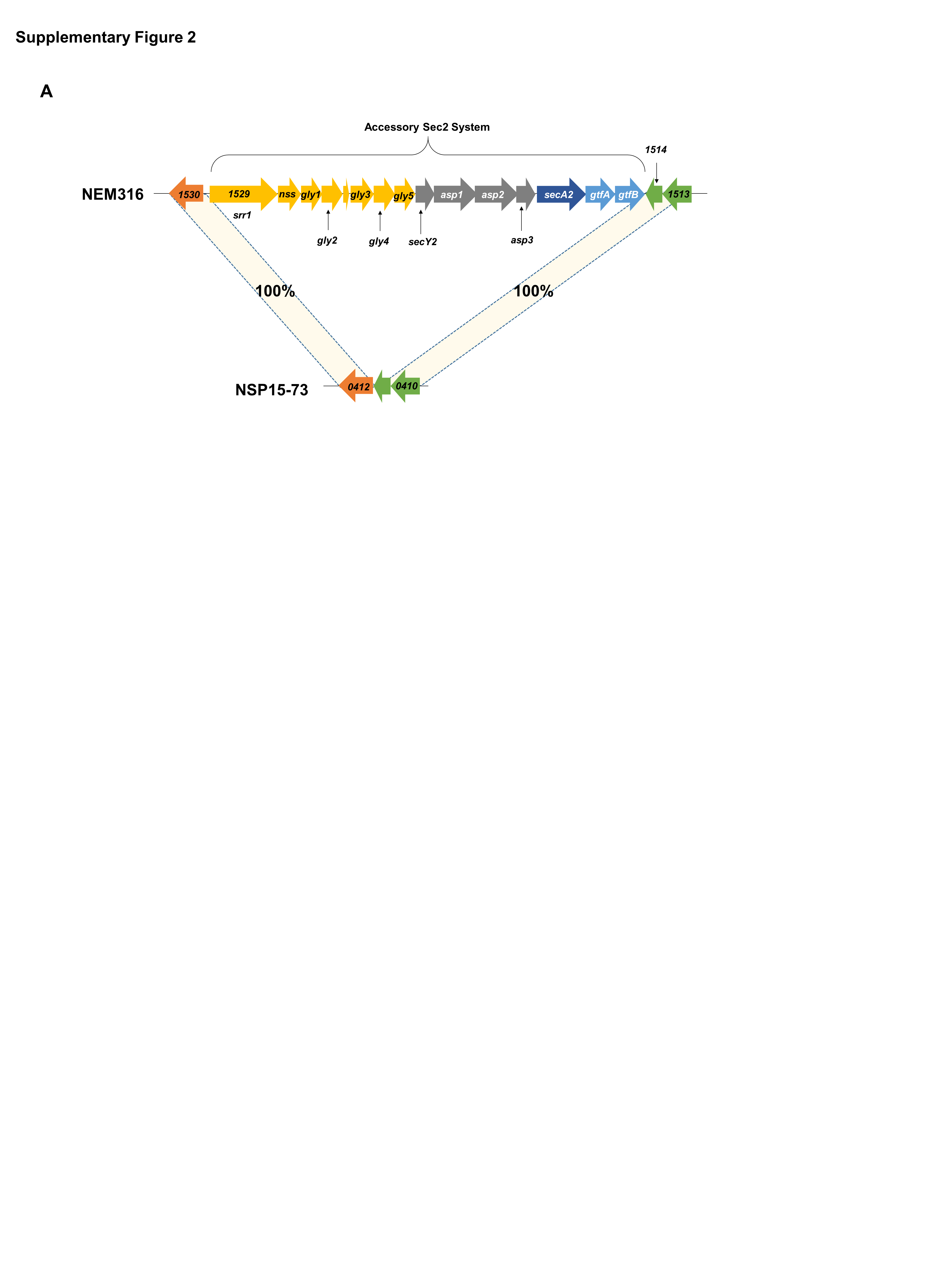

Supplement: FIG S2 [file msphere.00543-21-sf002.tif]

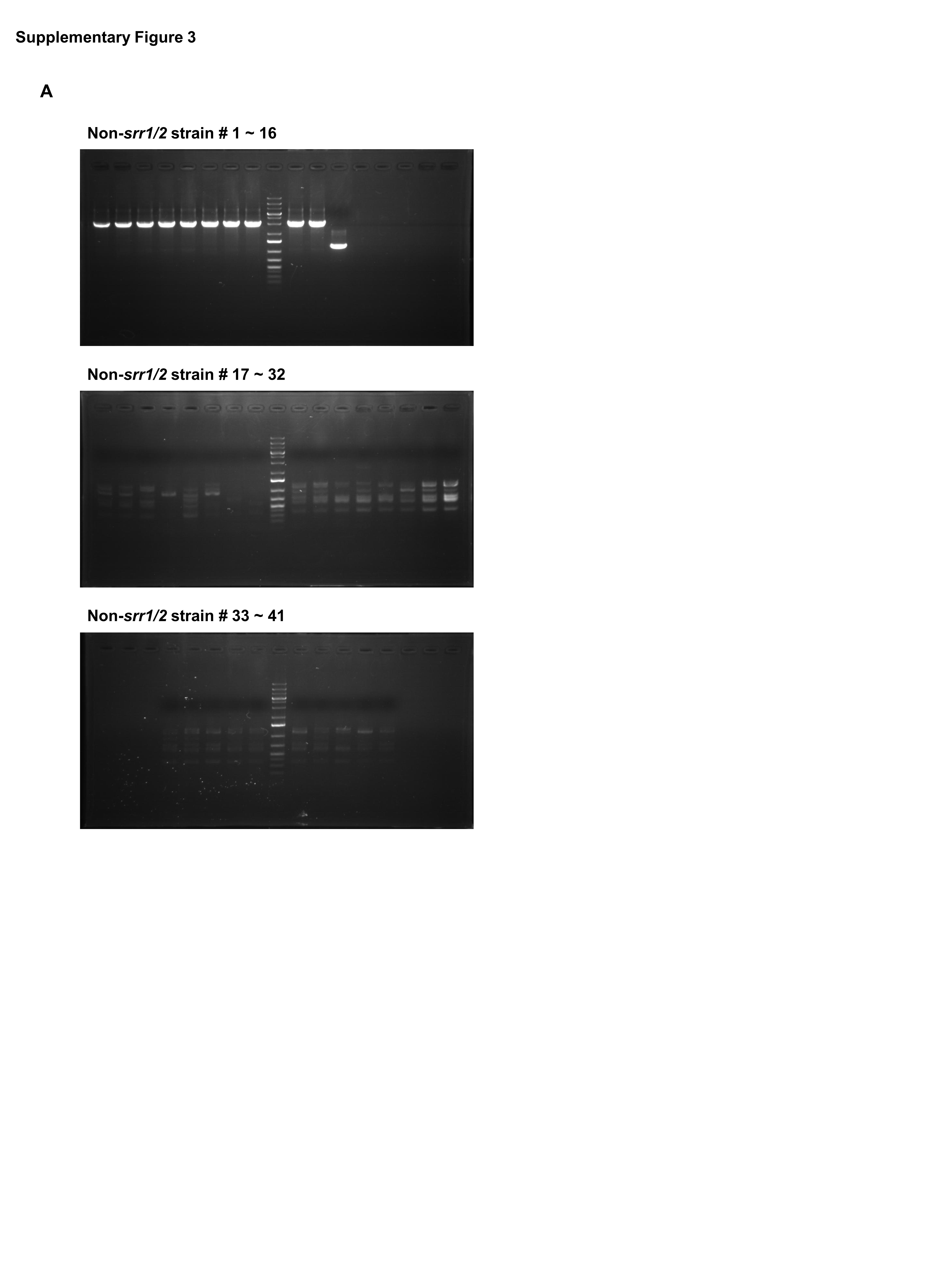

Supplement: FIG S3 [file msphere.00543-21-sf003.tif]
