# Supplementary material for: Molecular Characteristics of IS1216 Carrying Multidrug Resistance Gene Cluster in Serotype III/Sequence Type 19 Group B Streptococcus
Source: mSphere. 2021 Jul 28;6(4):e00543-21. doi: 10.1128/mSphere.00543-21 (PMC8386385; doi:10.1128/mSphere.00543-21)
Supplement: TABLE S1 [file msphere.00543-21-st001.docx]

| **Sample number** | **Strain** | **Serotype** | **MLST** | **Tissue origin** | **Age**  **(yr)** | **Sex** |
| --- | --- | --- | --- | --- | --- | --- |
| **1** | **S9968** | III | 19 | URINE, SPOT | 48 | Female |
| **2** | **S10039** | III | 19 | URINE, SPOT | 78 | Male |
| **3** | **S10124** | III | 19 | Vaginal, Swab | 51 | Female |
| **4** | **S10171** | III | 19 | URINE, SPOT | 25 | Female |
| **5** | **NSP14-81** | III | 19 | PUS, OPEN | 68 | Male |
| **6** | **NSP14-151** | III | 19 | Vaginal, Swab | 31 | Female |
| **7** | **NSP14-167** | III | 19 | Vaginal, Swab | 59 | Female |
| **8** | **NSP15-560** | III | 19 | Vaginal, Swab | 36 | Female |
| **9** | **NSP15-613** | III | 19 | URINE, SPOT | 57 | Female |
| **10** | **NSP16-136** | III | 19 | URINE, SPOT | 59 | Female |
| **11** | **GBS16-89** | III | 19 | Tissue (biopsy) | 33 | Male |
| **12** | **S10120** | III | 19 | URINE, SPOT | 44 | Female |
| **13** | **NSP14-66** | III | 19 | URINE, SPOT | 43 | Male |
| **14** | **NSP15-659** | III | 19 | URINE, SPOT | 84 | Female |
| **15** | **NSP16-31** | III | 19 | PUS, OPEN | 59 | Female |
| **16** | **GBS16-54** | III | 19 | Vaginal, Swab | 36 | Female |
| **17** | **S9928** | III | 27 | URINE, SPOT | 59 | Female |
| **18** | **S9981** | III | 27 | URINE, SPOT | 37 | Female |
| **19** | **S10048** | III | 27 | Vaginal, Swab | 37 | Female |
| **20** | **S10072** | III | 27 | URINE, SPOT | 76 | Female |
| **21** | **S10121** | III | 27 | URINE, SPOT | 48 | Female |
| **22** | **NSP14-76** | III | 27 | URINE, SPOT | 34 | Male |
| **23** | **NSP14-110** | III | 27 | URINE, SPOT | 64 | Female |
| **24** | **NSP14-149** | III | 27 | URINE, SPOT | 48 | Male |
| **25** | **NSP14-150** | III | 27 | URINE, SPOT | 54 | Male |
| **26** | **NSP14-161** | III | 27 | URINE, SPOT | 64 | Female |
| **27** | **NSP14-259** | III | 27 | URINE, SPOT | 37 | Male |
| **28** | **NSP15-73** | III | 27 | URINE, SPOT | 50 | Female |
| **29** | **NSP15-236** | III | 27 | URINE, SPOT | 30 | Female |
| **30** | **NSP15-531** | III | 27 | URINE, SPOT | 51 | Female |
| **31** | **NSP15-584** | III | 27 | URINE, SPOT | 34 | Female |
| **32** | **NSP15-635** | III | 27 | URINE, SPOT | 59 | Male |
| **33** | **NSP16-102** | III | 27 | Vaginal, Swab | 26 | Female |
| **34** | **GBS16-40** | III | 27 | URINE, SPOT | 57 | Female |
| **35** | **S10003** | III | 529 | URINE, SPOT | 24 | Female |
| **36** | **S10161** | III | 529 | URINE, SPOT | 34 | Female |
| **37** | **NSP14-28** | III | 529 | URINE, SPOT | 41 | Female |
| **38** | **NSP14-87** | III | 529 | Vaginal, Swab | 31 | Female |
| **39** | **NSP15-667** | III | 529 | Sputum | 84 | Female |
| **40** | **GBS16-17** | III | 529 | Tissue (biopsy) | 28 | Male |
| **41** | **NSP14-65** | III | 171 | URINE, SPOT | 0.33 | Female |

**Supplementary Table 1. Strain information of 41 non-*srr1/2* clinical isolates**

**^a^ N/A: Not available**
