# Supplementary material for: Molecular Characteristics of IS1216 Carrying Multidrug Resistance Gene Cluster in Serotype III/Sequence Type 19 Group B Streptococcus
Source: mSphere. 2021 Jul 28;6(4):e00543-21. doi: 10.1128/mSphere.00543-21 (PMC8386385; doi:10.1128/mSphere.00543-21)
Supplement: TABLE S2 [file msphere.00543-21-st002.docx]

**Supplementary Table 2. Genes in Site 1, 2, 3, and 4 cluster of S9968**

| **Site 1 cluster** |  |  |  |
| --- | --- | --- | --- |
| **Protein_ID** | **Start** | **End** | **Homologous protein** |
| S9968_1_00416 | 358767 | 359157 | hypothetical protein |
| S9968_1_00417 | 359159 | 359675 | LytTR family two-component system response regulator (*Streptococcus agalactiae*) |
| S9968_1_00418 | 359684 | 360428 | ABC transporter permease (*Streptococcus agalactiae*) |
| S9968_1_00419 | 360411 | 361281 | ABC transporter ATP-binding protein (*Streptococcus agalactiae*) |
| S9968_1_00420 | 361282 | 362248 | hypothetical protein |
| S9968_1_00421 | 362231 | 363041 | SagB/ThcOx family dehydrogenase (*Streptococcus sp*.) |
| S9968_1_00422 | 363053 | 364268 | YcaO-like family protein (*Streptococcus agalactiae*) |
| S9968_1_00423 | 364257 | 365130 | bacteriocin biosynthesis cyclodehydratase domain (*Streptococcus agalactiae*) |
| S9968_1_00424 | 365859 | 366216 | Tn5252 transposon protein (*Streptococcus agalactiae*) |
| S9968_1_00425 | 366215 | 366578 | Transposon protein (*Streptococcus agalactiae*) |
| S9968_1_00426 | 366570 | 366861 | Tn5253 hypothetical protein (*Streptococcus agalactiae*) |
| S9968_1_00427 | 366875 | 367700 | putative DNA replication protein (*Streptococcus agalactiae*) |
| S9968_1_00428 | 367708 | 367993 | DNA-binding protein (*Streptococcus anginosus*) |
| S9968_1_00429 | 368141 | 368660 | XRE family transcriptional regulator (*Streptococcus agalactiae*) |
| S9968_1_00430 | 368714 | 369869 | phage integrase family site specific recombinase (*Streptococcus agalactiae*) |
| **Site 2 cluster** |  |  |  |
| **Protein_ID** | **Start** | **End** | **Homologous protein** |
| S9968_1_00864 | 776223 | 777345 | integrase (*Streptococcus agalactiae*) |
| S9968_1_00866 | 778109 | 779393 | ISLre2 family transposase (Streptococcus) |
| S9968_1_00867 | 779423 | 779831 | hypothetical protein |
| S9968_1_00868 | 779967 | 781608 | relaxase/mobilization nuclease domain protein (S. agalactiae CNCTC 10/84) |
| S9968_1_00869 | 781579 | 781954 | plasmid mobilization relaxosome protein MobC (S. agalactiae) |
| S9968_1_00870 | 781956 | 782517 | hypothetical protein |
| S9968_1_00871 | 782859 | 783159 | hypothetical protein |
| S9968_1_00872 | 783210 | 786450 | DNA primase (*Streptococcus agalactiae*) |
| S9968_1_00873 | 786451 | 786817 | hypothetical protein |
| S9968_1_00874 | 786858 | 788904 | conjugal transfer protein TraG (*Streptococcus agalactiae*) |
| S9968_1_00875 | 788903 | 789395 | conjugative transposon protein (*Streptococcus agalactiae*) |
| S9968_1_00876 | 789416 | 790202 | hypothetical protein |
| S9968_1_00877 | 790201 | 790474 | hypothetical protein |
| S9968_1_00878 | 790493 | 791099 | parvulin-like peptidyl-prolyl isomerase (*Streptococcus agalactiae*) |
| S9968_1_00879 | 791119 | 793810 | amidase (*Streptococcus agalactiae* CCUG 37742) |
| S9968_1_00880 | 793844 | 794171 | hypothetical protein |
| S9968_1_00881 | 794194 | 794788 | hypothetical protein |
| S9968_1_00882 | 794780 | 797141 | AAA family ATPase (*Streptococcus agalactiae*) |
| S9968_1_00883 | 797121 | 797481 | hypothetical protein |
| S9968_1_00884 | 797542 | 798001 | hypothetical protein |
| S9968_1_00885 | 797993 | 798290 | hypothetical protein |
| S9968_1_00886 | 798326 | 799079 | hypothetical protein |
| S9968_1_00887 | 799129 | 799711 | hypothetical protein |
| S9968_1_00888 | 799815 | 800043 | hypothetical protein |
| S9968_1_00889 | 800325 | 803127 | LPXTG cell wall anchor domain-containing protein (Streptococcus sp.) |
| S9968_1_00890 | 803179 | 803902 | LPXTG cell wall anchor domain-containing protein (*Streptococcus agalactiae*) |
| S9968_1_00891 | 803921 | 806183 | LPXTG cell wall anchor domain-containing protein (*Streptococcus agalactiae*) |
| S9968_1_00892 | 806199 | 806499 | hypothetical protein |
| S9968_1_00893 | 807075 | 807216 | hypothetical protein |
| S9968_1_00894 | 807212 | 807902 | hypothetical protein |
| S9968_1_00895 | 807895 | 808930 | replication initiator protein A [*Streptococcus agalactiae*] |
| S9968_1_00896 | 808931 | 809096 | hypothetical protein |
| S9968_1_00897 | 809202 | 809379 | hypothetical protein |
| S9968_1_00898 | 809375 | 809708 | hypothetical protein |
| S9968_1_00899 | 809747 | 810101 | XRE family transcriptional regulator [*Streptococcus pyogenes*] |
| S9968_1_00900 | 810123 | 810291 | hypothetical protein |
| S9968_1_00901 | 810268 | 810526 | hypothetical protein |
| S9968_1_00902 | 810522 | 810699 | hypothetical protein |
| S9968_1_00903 | 810709 | 811081 | hypothetical protein |
| **Site 3 cluster** |  |  |  |
| **Protein_ID** | **Start** | **End** | **Homologous protein** |
| S9968_1_00864 | 776223 | 777345 | integrase (*Streptococcus agalactiae*) |
| S9968_1_00866 | 778109 | 779393 | ISLre2 family transposase (Streptococcus) |
| S9968_1_00867 | 779423 | 779831 | hypothetical protein |
| S9968_1_00868 | 779967 | 781608 | relaxase/mobilization nuclease domain protein (S. agalactiae CNCTC 10/84) |
| S9968_1_00869 | 781579 | 781954 | plasmid mobilization relaxosome protein MobC (S. agalactiae) |
| S9968_1_00870 | 781956 | 782517 | hypothetical protein |
| S9968_1_00871 | 782859 | 783159 | hypothetical protein |
| S9968_1_00872 | 783210 | 786450 | DNA primase (*Streptococcus agalactiae*) |
| S9968_1_00873 | 786451 | 786817 | hypothetical protein |
| S9968_1_00874 | 786858 | 788904 | conjugal transfer protein TraG (*Streptococcus agalactiae*) |
| S9968_1_00875 | 788903 | 789395 | conjugative transposon protein (*Streptococcus agalactiae*) |
| S9968_1_00876 | 789416 | 790202 | hypothetical protein |
| S9968_1_00877 | 790201 | 790474 | hypothetical protein |
| S9968_1_00878 | 790493 | 791099 | parvulin-like peptidyl-prolyl isomerase (*Streptococcus agalactiae*) |
| S9968_1_00879 | 791119 | 793810 | amidase (*Streptococcus agalactiae* CCUG 37742) |
| S9968_1_00880 | 793844 | 794171 | hypothetical protein |
| S9968_1_00881 | 794194 | 794788 | hypothetical protein |
| S9968_1_00882 | 794780 | 797141 | AAA family ATPase (*Streptococcus agalactiae*) |
| S9968_1_00883 | 797121 | 797481 | hypothetical protein |
| S9968_1_00884 | 797542 | 798001 | hypothetical protein |
| S9968_1_00885 | 797993 | 798290 | hypothetical protein |
| S9968_1_00886 | 798326 | 799079 | hypothetical protein |
| S9968_1_00887 | 799129 | 799711 | hypothetical protein |
| S9968_1_00888 | 799815 | 800043 | hypothetical protein |
| S9968_1_00889 | 800325 | 803127 | LPXTG cell wall anchor domain-containing protein (Streptococcus sp.) |
| S9968_1_00890 | 803179 | 803902 | LPXTG cell wall anchor domain-containing protein (*Streptococcus agalactiae*) |
| S9968_1_00891 | 803921 | 806183 | LPXTG cell wall anchor domain-containing protein (*Streptococcus agalactiae*) |
| S9968_1_00892 | 806199 | 806499 | hypothetical protein |
| S9968_1_00893 | 807075 | 807216 | hypothetical protein |
| S9968_1_00894 | 807212 | 807902 | hypothetical protein |
| S9968_1_00895 | 807895 | 808930 | replication initiator protein A [*Streptococcus agalactiae*] |
| S9968_1_00896 | 808931 | 809096 | hypothetical protein |
| S9968_1_00897 | 809202 | 809379 | hypothetical protein |
| S9968_1_00898 | 809375 | 809708 | hypothetical protein |
| S9968_1_00899 | 809747 | 810101 | XRE family transcriptional regulator [*Streptococcus pyogenes*] |
| S9968_1_00900 | 810123 | 810291 | hypothetical protein |
| S9968_1_00901 | 810268 | 810526 | hypothetical protein |
| S9968_1_00902 | 810522 | 810699 | hypothetical protein |
| S9968_1_00903 | 810709 | 811081 | hypothetical protein |
| **Site 4 cluster** |  |  |  |
| **Protein_ID** | **Start** | **End** | **Homologous protein** |
| S9968_1_02230 | 2177544 | 2178225 | IS6-like element IS1216 family transposase |
| S9968_1_02231 | 2178247 | 2178799 | AadE: aminoglycoside 6-adenyltransferase |
| S9968_1_02232 | 2178890 | 2178980 | hypothetical protein |
| S9968_1_02233 | 2179064 | 2180504 | Aac/Aph: aminoglycoside O-phosphotransferase APH(2'')-Ia |
| S9968_1_02234 | 2180504 | 2181026 | GNAT family N-acetyltransferase |
| S9968_1_02235 | 2181058 | 2181793 | class I SAM-dependent methyltransferase |
| S9968_1_02236 | 2181773 | 2182643 | DNA polymerase, nucleotidyltransferase |
| S9968_1_02237 | 2182657 | 2182882 | XRE family transcriptional regulator |
| S9968_1_02238 | 2183381 | 2184461 | Tnp: ISL3 family Transposase |
| S9968_1_02239 | 2184909 | 2185713 | Lnu(B): Lincosamide nucleotidyltransferase |
| S9968_1_02240 | 2185766 | 2187251 | Lsa(E); ABC-F type ribosomal protection protein |
| S9968_1_02241 | 2187693 | 2188179 | DNA recombinase (*Enterococcus faecalis* TX2137) |
| S9968_1_02242 | 2188339 | 2188708 | hypothetical protein |
| S9968_1_02243 | 2188730 | 2189411 | IS6-like element IS1216 family transposase |
| S9968_1_02244 | 2189464 | 2189683 | hypothetical protein |
| S9968_1_02245 | 2189695 | 2193517 | prophage minor structural protein (*Streptococcus agalactiae*) |
| S9968_1_02246 | 2193507 | 2195016 | phage tail protein (*Streptococcus agalactiae*) |
| S9968_1_02247 | 2195027 | 2198951 | phage tail tape measure protein (*Streptococcus agalactiae*) |
| S9968_1_02248 | 2199177 | 2199480 | phage tail protein (*Streptococcus agalactiae*) |
| S9968_1_02249 | 2199490 | 2200096 | phage tail protein (*Streptococcus agalactiae*) |
| S9968_1_02250 | 2200110 | 2200491 | hypothetical protein |
| S9968_1_02251 | 2200483 | 2200876 | hypothetical protein |
| S9968_1_00001 | 106 | 256 | hypothetical protein |
| S9968_1_00002 | 227 | 521 | phage gp6-like connector protein (*Streptococcus agalactiae*) |
| S9968_1_00003 | 524 | 1685 | phage major capsid protein (*Streptococcus agalactiae*) |
| S9968_1_00004 | 1706 | 2438 | ClpP, Clp protease (*Streptococcus agalactiae*) |
| S9968_1_00005 | 2418 | 3567 | phage portal protein (*Streptococcus agalactiae*) |
| S9968_1_00006 | 3638 | 3962 | hypothetical protein |
| S9968_1_00007 | 3972 | 5616 | putative phage terminase large subunit (*Streptococcus agalactiae*) |
