# Supplementary material for: Molecular Characteristics of IS1216 Carrying Multidrug Resistance Gene Cluster in Serotype III/Sequence Type 19 Group B Streptococcus
Source: mSphere. 2021 Jul 28;6(4):e00543-21. doi: 10.1128/mSphere.00543-21 (PMC8386385; doi:10.1128/mSphere.00543-21)
Supplement: TABLE S3 [file msphere.00543-21-st003.docx]

**Supplementary Table 3. Primers for multiplex polymerase chain reaction serotyping**

| **Primer** | **Sequence (5**ʹ **to 3**ʹ**)** | **Description** |
| --- | --- | --- |
| **Ia-F** | GGT CAG ACT GGA TTA ATG GTA TGC | *cps1aH* |
| **Ia-R** | GTA GAA ATA GCC TAT ATA CGT TGA ATG C |  |
| **Ib*-*F** | TAA ACG AGA ATG GAA TAT CAC AAA CC | *cps1bJ/K* |
| **Ib-R** | GAA TTA ACT TCA ATC CCT AAA CAA TAT CG |  |
| **II-F** | GCT TCA GTA AGT ATT GTA AGA CGA TAG | *cps2K* |
| **II-R** | TTC TCT AGG AAA TCA AAT AAT TCT ATA GGG |  |
| **III-F** | TCC GTA CTA CAA CAG ACT CAT CC | *cps1a/2/3I* |
| **III-R** | AGT AAC CGT CCA TAC ATT CTA TAA GC |  |
| **IV-F** | GGT GGT AAT CCT AAG AGT GAA CTG T | *cps4N* |
| **IV-R** | AAG AAA TCT CTT GTG CGG AT |  |
| **V-F** | GAG GCC AAT CAG TTG CAC GTA A | *cps5O* |
| **V-R** | AAC CTT CTC CTT CAC ACT AAT CCT |  |
| **VI-F** | GGA CTT GAG ATG GCA GAA GGT GAA | *cps6I* |
| **VI-R** | CTG TCG GAC TAT CCT GAT GAA TCT C |  |
| **VII-F** | CCT GGA GAG AAC AAT GTC CAG AT | *cps7M* |
| **VII-R** | GCT GGT CGT GAT TTC TAC ACA |  |
| **VIII-F** | AGG TCA ACC ACT ATA TAG CGA | *cps8J* |
| **VIII-R** | TCT TCA AAT TCC GCT GAC TT |  |
| **IX-F** | CTG TAA TTG GAG GAA TGT GGA TCG | *cps9I* |
| **IX-R** | AAT CAT CTT CAT AAT TTA TCT CCC ATT |  |

^a^PCR reactions were as follows: one cycle at 95°C for 5 min, 30 cycles of 95°C for 20 sec, 56.2°C for 20 sec, and 72°C for 1.5 min, and 1 cycle of 72°C for 5 min
