# Supplementary material for: Molecular Characteristics of IS1216 Carrying Multidrug Resistance Gene Cluster in Serotype III/Sequence Type 19 Group B Streptococcus
Source: mSphere. 2021 Jul 28;6(4):e00543-21. doi: 10.1128/mSphere.00543-21 (PMC8386385; doi:10.1128/mSphere.00543-21)
Supplement: TABLE S4 [file msphere.00543-21-st004.docx]

**Supplementary Table 4. Primers used for the PCR detection of *srr1/2* genes and IS1216**

| **Primer** | **Sequence (5**ʹ **to 3**ʹ**)** | **Description** |
| --- | --- | --- |
| **3084** | CGG GAT CCG AAG AAG TGT CTG ATA CTG AAA TG | *srr1*^a^ |
| **5084** | AAG CGG CCG CTT ATA AAA GTT TAA TTT CGG CAT TC |  |
| **3085** | CGG GAT CCA CTT TAC CAG CAG CAT TAA TC | *srr2*^a^ |
| **5078** | GTG CGG CCG CAT TCA GAT GAG TAA GTA TGA TTA TG |  |
| **3501** | TGA TGC AAA AGC CGG AGG AT | IS1216 diagnosis PCR^b^ |
| **5501** | AAA GCA AAT GCG GGG GTA GA |  |
| ^a^ PCR cycles were as follows: one cycle at 95°C for 5 min, 30 cycles of 95°C for 20 sec, 56.2°C for 20 sec, and 72°C for 1.5 min, and 1 cycle of 72°C for 5 min.  ^b^ PCR cycles were as follows: one cycle at 95°C for 5 min, 30 cycles of 95°C for 20 sec, 56.2°C for 20 sec, and 72°C for 3.5 min, and 1 cycle of 72°C for 5 min. | | |
