# Supplementary material for: Molecular Characteristics of IS1216 Carrying Multidrug Resistance Gene Cluster in Serotype III/Sequence Type 19 Group B Streptococcus
Source: mSphere. 2021 Jul 28;6(4):e00543-21. doi: 10.1128/mSphere.00543-21 (PMC8386385; doi:10.1128/mSphere.00543-21)
Supplement: TABLE S5 [file msphere.00543-21-st005.docx]

**Supplementary Table 5. Primers for Multi-Locus Sequencing Typing**

| **Primer** | **Sequence (5**ʹ **to 3**ʹ**)** | **Description** |
| --- | --- | --- |
| ***adhP*-F** | GTT GGT CAT GGT GAA GCA CT | *adhP* PCR |
| ***adhP*-R** | ACT GTA CCT CCA GCA CGA AC |  |
| ***adhP*-S-F** | GGT GTG TGC CAT ACT GAT TT | *adhP* sequencing |
| ***adhP*-S-R** | ACA GCA GTC ACA ACC ACT CC |  |
| ***pheS*-F** | GAT TAA GGA GTA GTG GCA CG | *pheS* PCR |
| ***pheS*-R** | TTG AGA TCG CCC ATT GAA AT |  |
| ***pheS*-S-F** | ATA TCA ACT CAA GAA AAG CT | *pheS* sequencing |
| ***pheS*-S-R** | TGA TGG AAT TGA TGG CTA TG |  |
| ***atr*-F** | CGA TTC TCT CAG CTT TGT TA | *atr* PCR |
| ***atr*-R** | AAG AAA TCT CTT GTG CGG AT |  |
| ***atr*-S-F** | ATG GTT GAG CCA ATT ATT TC | *atr* sequencing |
| ***atr-*S-R** | CCT TGC TCA ACA ATA ATG CC |  |
| ***glnA*-F** | CCG GCT ACA GAT GAA CAA TT | *glnA* PCR |
| ***glnA*-R** | CCG ATA ATT GCC ATT CCA CG |  |
| ***glnA*-S-F** | AAT AAA GCA ATG TTT GAT GG | *glnA* sequencing |
| ***glnA-*S-R** | GCA TTG TTC CCT TCA TTA TC |  |
| ***sdhA*-F** | AGA GCA AGC TAA TAG CCA AC | *sdhA* PCR |
| ***sdhA*-R** | ATA TCA GCA GCA ACA AGT GC |  |
| ***sdhA*-S-F** | AAC ATA GCA GAG CTC ATG AT | *sdhA* sequencing |
| ***sdhA-*S-R** | GGG ACT TCA ACT AAA CCT GC |  |
| ***glcK*-F** | CTC GGA GGA ACG ACC ATT AA | *glcK* PCR |
| ***glcK*-R** | CTT GTA ACA GTA TCA CCG TT |  |
| ***glcK*-S-F** | GGT ATC TTG ACG CTT GAG GG | *glcK* sequencing |
| ***glcK-*S-R** | ATC GCT GCT TTA ATG GCA GA |  |
| ***tkt*-F** | CCA GGC TTT GAT TTA GTT GA | *tkt* PCR |
| ***tkt*-R** | AAT AGC TTG TTG GCT TGA AA |  |
| ***tkt*-S-F** | ACA CTT CAT GGT GAT GGT TG | *tkt* sequencing |
| ***tkt-*S-R** | TGA CCT AGG TCA TGA GCT TT |  |
| ^a^ PCR reactions were as follows: one cycle at 95°C for 5 min, 30 cycles of 95°C for 20 sec, 56.2°C for 20 sec, and 72°C for 1.5 min, and 1 cycle of 72°C for 5 min. | | |
