# Supplementary material for: Molecular Characteristics of IS1216 Carrying Multidrug Resistance Gene Cluster in Serotype III/Sequence Type 19 Group B Streptococcus
Source: mSphere. 2021 Jul 28;6(4):e00543-21. doi: 10.1128/mSphere.00543-21 (PMC8386385; doi:10.1128/mSphere.00543-21)
Supplement: TABLE S6 [file msphere.00543-21-st006.docx]

**Supplementary Table 6. GBS accession numbers used for genome comparison**

| **Name** | **Serotype** | **MLST** | **Accession No** |
| --- | --- | --- | --- |
| SG-M50 | III | ST283 | CP021865.1 |
| SG-M29 | III | ST283 | CP021866.1 |
| SGEHI2015-95 | III | ST283 | CP025028.1 |
| SG-M163 | III | ST283 | CP021863.1 |
| SG-M1 | III | ST283 | CP012419.2 |
| CU GBS 98 | III | ST283 | NZ_CP010875.1 |
| CU GBS 08 | III | ST283 | NZ_CP010874.1 |
| FWL1402 | III | ST739 | NZ_CP016391.1 |
| SGEHI2015-107 | III | ST283 | CP025027.1 |
| S73 | III | ST283 | KY635949 |
| SGEHI2015-113 | III | ST283 | CP025026.1 |
| NEM316 | III | ST23 | AL732656 |
| HU-GS5823 | III | ST335 | AP018935 |
| Sag158 | III | ST19 | NZ_CP019979.1 |
| S9968 | III | ST19 | SAMN15246708 |
| H002 | III | ST19 | CP011329. |
| NCTC8184 | III | ST17 | GCF_900636375 |
| BM110 | III | ST17 | GCA_900155855.1 |
| 874391 | III | ST17 | CP022537 |
| NGBS128 | III | ST17 | CP012480 |
| COH1 | III | ST17 | HG93945 |
